# Sirtuins at the Interface of Glucose Metabolism, Diabetes, and Heart Failure: Metabolic Sensing in Cardiometabolic Disease

**DOI:** 10.3390/ijms27135780

**Published:** 2026-06-26

**Authors:** Jan Krekora, Jarosław Drożdż, Elzbieta Pawlowska, Janusz Blasiak

**Affiliations:** 1 2nd Department of Cardiology, Medical University of Lodz, 92-213 Lodz, Poland; ejkrekora@op.pl (J.K.); jaroslaw.drozdz@umed.lodz.pl (J.D.); 2 Department of Pediatric Dentistry, Medical University of Lodz, 92-213 Lodz, Poland; elzbieta.pawlowska@umed.lodz.pl; 3 Faculty of Medicine, Mazovian University in Plock, 09-240 Plock, Poland

**Keywords:** sirtuins, heart failure, glucose, diabetes, metabolism, energetic efficiency, NAD^+^, mitochondrial impairment

## Abstract

Heart failure (HF) in the setting of diabetes represents a distinct cardiometabolic phenotype characterized by profound disturbances in myocardial glucose metabolism, mitochondrial function, and energetic efficiency. Growing evidence indicates that sirtuins, a family of nicotinamide adenine dinucleotide (NAD^+^)-dependent deacylases, play a central role in coordinating glucose utilization, oxidative metabolism, and stress responses in the heart. Findings from genetically modified animal models and cardiomyocyte studies demonstrate that sirtuin impairment, often driven by NAD^+^ depletion and redox imbalance, further suppresses metabolic activity and promotes metabolic inflexibility, whereas restoration of NAD^+^ availability or sirtuin activity improves mitochondrial efficiency and metabolic coordination. Human studies, including analyses of myocardial tissue and circulating biomarkers, provide supportive but largely associative evidence, highlighting a substantial translational gap. In this review, we synthesize experimental and clinical data linking sirtuin signaling to the metabolic remodeling observed in diabetic HF, with particular emphasis on glycolysis–oxidation uncoupling, pyruvate dehydrogenase regulation, and mitochondrial dysfunction. We critically discuss context-dependent effects, apparent contradictions, and current limitations of the field, emphasizing differences between diabetic and non-diabetic HF, as well as phenotype- and stage-specific considerations. Finally, we explore therapeutic implications and outstanding questions, positioning the NAD^+^–sirtuin axis as a unifying mechanistic framework that links systemic metabolic disease to cardiac energetic failure and underscores the potential for metabolism-informed, precision strategies in diabetic HF.

## 1. Introduction

Despite major advances in clinical management, heart failure (HF) remains a leading cause of morbidity and mortality worldwide, underscoring the limitations of current therapeutic strategies and the need for deeper mechanistic insight [1]. The persistent residual risk observed even with optimal guideline-directed therapy highlights that key disease-driving processes operate at the molecular and cellular levels, beyond hemodynamic correction and neurohormonal blockade [2]. Consequently, molecular research has become essential for identifying the fundamental pathways that govern disease initiation, progression, and therapeutic response in HF.

Conceptual understanding of HF has evolved markedly over the past decades, shifting from a predominantly hemodynamic model to a neurohormonal paradigm and, more recently, to a metabolic view of the disease [3]. While early frameworks focused on impaired cardiac output and ventricular loading, subsequent recognition of maladaptive neurohormonal activation established HF as a systemic disorder amenable to pharmacological modulation. However, accumulating experimental and clinical evidence has revealed that the failing myocardium is characterized by profound disturbances in energy metabolism that precede or accompany the heart’s structural dysfunction [4]. The heart has since been recognized as a metabolically flexible organ whose capacity to adapt substrate utilization becomes progressively constrained in HF, leading to impaired ATP generation, mitochondrial dysfunction, and redox imbalance [5]. This metabolic perspective integrates alterations in fatty acid and glucose utilization, mitochondrial efficiency, and cellular energy sensing, positioning energetic failure as both a driver and a consequence of disease progression. Importantly, this shift has reframed HF not merely as a disorder of pump function or signaling excess, but as a state of compromised metabolic homeostasis, thereby opening new avenues for mechanistic investigation and therapeutic intervention. HF has different phenotypes, including HF with preserved and reduced left ventricular ejection fraction (HFpEF and HFrEF, respectively), which may differ substantially in cardiac energy metabolism [6].

Diabetes is a major and independent risk factor for cardiovascular disease (CVD), substantially increasing the incidence of coronary artery disease, HF, stroke, and cardiovascular mortality [7]. Chronic hyperglycemia, insulin resistance, and associated metabolic disturbances promote endothelial dysfunction, inflammation, oxidative stress, and adverse cardiac remodeling, thereby actively driving cardiovascular pathology rather than merely accompanying it [8]. Consequently, diabetes now represents one of the most powerful modifiers of CVD phenotype, progression, and therapeutic response, positioning cardiometabolic disease at the center of contemporary cardiovascular research. The diabetes–CVD association also includes HF [9].

Sirtuins (SIRTs) comprise a conserved, seven-member family of nicotinamide adenine dinucleotide (NAD^+^)-dependent deacylases and ADP-ribosyltransferases that regulate gene expression, metabolism, and stress responses across multiple cellular compartments [10]. Sirtuins also play a prominent role in antioxidant and redox signaling [11]. In the context of diabetes and CVD, sirtuins have emerged as key molecular regulators linking metabolic status to cardiovascular pathology [12]. As metabolic sensors, they modulate pathways central to diabetic cardiovascular injury, including glucose utilization, mitochondrial function, oxidative stress, and inflammation, thereby providing a mechanistic link by which diabetic metabolic disturbances translate into adverse cardiovascular remodeling and dysfunction [13,14].

Chronic hyperglycemia and insulin resistance drive sustained alterations in myocardial glucose handling, cause NAD^+^ depletion, and increase oxidative and inflammatory stress, thereby compromising sirtuin activity even when their expression remains unchanged [15]. This is especially evident in diabetic cardiomyopathy, where impaired sirtuin-mediated regulation of glycolytic flux, glucose oxidation, mitochondrial protein acetylation, and antioxidant defenses contribute to energetic inefficiency and disease progression [16]. Glucose oxidation is used here as a general term referring to the mitochondrial metabolism of glucose-derived pyruvate via pyruvate dehydrogenase (PDH), the tricarboxylic acid cycle, and oxidative phosphorylation, leading to ATP production and CO_2_ generation. Accordingly, the convergence of diabetes, altered glucose metabolism, and sirtuin dysfunction may provide a unifying molecular framework for understanding metabolic inflexibility, mitochondrial failure, and maladaptive remodeling in HF, particularly in the HFpEF phenotype, which is tightly coupled to cardiometabolic disease [17].

Therefore, accumulating evidence positions HF as a disease of disrupted metabolic homeostasis, in which altered glucose handling, redox imbalance, and mitochondrial dysfunction play central pathogenic roles, particularly in the setting of diabetes. Sirtuins, as NAD^+^-dependent metabolic and stress sensors, link glucose metabolism to transcriptional regulation, mitochondrial function, and adaptive cardiac remodeling. The rationale for this narrative/perspective review, therefore, lies in the need to examine integratively how sirtuin signaling is shaped by diabetic and HF-associated metabolic perturbations and how this interaction contributes to disease initiation and progression. Accordingly, the aim of this review is to critically synthesize current experimental and clinical evidence on sirtuin-mediated regulation of cardiac glucose metabolism in HF, identify unresolved questions and conceptual gaps, and outline future research directions that may inform metabolically targeted and precision-based therapeutic strategies.

## 2. Glucose Metabolism Remodeling in Heart Failure

The healthy adult heart is a metabolically flexible organ that relies predominantly on fatty acid (FA) oxidation while retaining the capacity to dynamically increase glucose utilization in response to workload, substrate availability, and hormonal cues [18]. In HF, this flexibility is progressively lost, and myocardial glucose metabolism undergoes coordinated remodeling that reflects both adaptive responses to energetic stress and maladaptive processes that drive HF progression. Central to this remodeling is altered sensing of cellular energy and redox state, in which SIRTs play a critical role by coupling substrate flux to transcriptional, enzymatic, and mitochondrial regulation [19].

### 2.1. Substrate Switching from Fatty Acids to Glucose

A shift from FA oxidation toward increased glucose utilization is among the earliest metabolic changes observed in HF [20]. This substrate switch is initially adaptive because glucose oxidation yields more ATP per molecule of oxygen than FA oxidation, thereby improving energetic efficiency under ischemic or low-oxygen conditions [21]. Consequently, increased glucose uptake and glycolytic flux are commonly observed in hypertrophied and failing hearts [22]. At the molecular level, this transition is accompanied by changes in NAD^+^ availability and the acetylation status of metabolic regulators, positioning sirtuins to sense and modulate this substrate shift [23]. However, this mechanism may not generalize to HFpEF, in which FA oxidation remains active or even increases, while glucose oxidation becomes impaired [24]. Nuclear and cytosolic sirtuins, such as SIRT1 and SIRT6, respond to altered glucose flux by modulating transcriptional programs through NAD^+^-dependent deacetylation rather than direct DNA binding [25]. Specifically, SIRT1 deacetylates key transcriptional regulators such as PGC-1α, FOXO proteins, p53, and NF-κB, thereby promoting mitochondrial biogenesis, antioxidant defense, and metabolic adaptation [26]. SIRT6, in turn, represses glycolytic gene expression via deacetylation of histones at HIF-1α target promoters, limiting glycolytic flux. In mitochondria, sirtuins primarily regulate metabolism through post-translational modification of metabolic enzymes and redox regulators. SIRT3 enhances oxidative phosphorylation (OXPHOS) and the detoxification of reactive oxygen and nitrogen species (RONS) via deacetylation of electron transport chain (ETC) components and antioxidant enzymes, such as manganese superoxide dismutase (MnSOD), while also modulating key tricarboxylic acid (TCA) cycle enzymes [27]. SIRT4, initially characterized for its ADP-ribosylation-mediated inhibition of glutamate dehydrogenase (GDH), is now recognized as a broader regulator of mitochondrial metabolism, exerting lipoamidase, deacylase, and regulatory functions that influence amino acid catabolism, insulin secretion, and fatty acid oxidation [28]. SIRT4-mediated control of GDH, along with reversible acylation, including acetylation and succinylation, highlights a complex regulatory layer that governs anaplerotic flux and metabolic homeostasis, with implications for metabolic diseases [29]. SIRT5 further contributes by dynamically regulating lysine acylation states, such as desuccinylation, demalonylation, and deglutarylation of a series of metabolic enzymes involved in the TCA cycle, fatty acid oxidation, and ammonia detoxification pathways, thereby fine-tuning metabolic flux and redox balance [30,31]. Collectively, these mechanisms illustrate a coordinated and compartment-specific network in which sirtuins modulate both transcriptional and enzymatic layers of control, integrating nutrient status with energy metabolism and mitochondrial functions. As HF progresses, however, the adaptive nature of substrate switching weakens, and sirtuin-mediated metabolic control becomes increasingly compromised [32].

### 2.2. Uncoupling of Glycolysis and Glucose Oxidation

A hallmark of metabolic remodeling in HF is the uncoupling of enhanced glycolysis from impaired mitochondrial glucose oxidation [33]. Although glycolytic flux is often increased, pyruvate entry into the TCA cycle via PDH is frequently reduced due to PDH inhibition, mitochondrial dysfunction, and altered signaling [34]. This mismatch diverts pyruvate toward lactate production rather than complete oxidation. Sirtuins are implicated in this process through a bidirectional interplay with mitochondrial metabolism, encompassing regulation of PDH activity, mitochondrial protein acylation, and redox balance [35]. While mitochondrial sirtuins (e.g., SIRT3) deacetylate and activate components of the PDH complex and related metabolic enzymes, PDH activity itself generates acetyl-CoA, the principal acetyl donor, and reduces NAD^+^ to NADH, thereby opposing deacetylation and modulating sirtuin activity [36,37]. Thus, PDH and mitochondrial sirtuins form a tightly coupled system in which substrate availability and redox state dynamically regulate post-translational modification of metabolic enzymes. This balance may be further adjusted under physiological conditions such as circadian or diurnal metabolic transitions [38,39,40]. In particular, mitochondrial sirtuins do not directly sense the NADH/NAD^+^ ratio; rather, as NAD^+^-dependent enzymes, their activity is governed by NAD^+^ availability, which reflects the cellular redox state and metabolic conditions [41]. Through this mechanism, they regulate the post-translational modification of enzymes involved in glucose oxidation and mitochondrial energy metabolism. Impaired sirtuin activity, therefore, contributes to sustained uncoupling of glycolysis from oxidation, supporting energetic inefficiency and metabolic inflexibility in HF.

**Figure 1:**
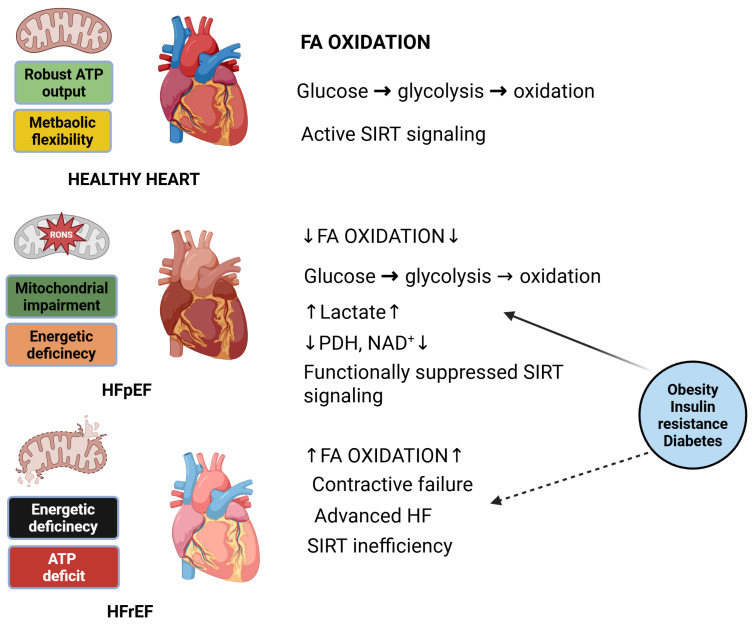
Glucose metabolism remodeling in heart failure (HF). The healthy heart exhibits metabolic flexibility, with efficient coupling of glycolysis to mitochondrial glucose oxidation, while fatty acid oxidation remains the dominant source of ATP. In HF with preserved ejection fraction (HFpEF), cardiometabolic stress increases glycolytic flux while impairing glucose oxidation, leading to oxidative stress, increased production of mitochondrial reactive oxygen and nitrogen species (RONS), and energetic inefficiency despite preserved systolic function and moderately impaired mitochondrial efficiency. HFpEF frequently coexists with obesity, insulin resistance, and diabetes, which play a central pathogenic role. In HF with reduced ejection fraction (HFrEF), advanced mitochondrial dysfunction, marked inhibition of pyruvate dehydrogenase (PDH), and nicotinamide adenine dinucleotide (NAD^+^) depletion result in profound uncoupling of glucose metabolism and energetic failure, accompanied by severe mitochondrial impairment. Progressive loss of sirtuin-mediated metabolic coordination parallels disease severity. Obesity, insulin resistance, and diabetes are common but non-obligatory comorbidities in HFrEF that exacerbate metabolic dysfunction. Differences in heart coloration are illustrative and do not imply discrete biological boundaries. Some mechanisms shown in the figure are explained in more detail in the main text. Created in BioRender. Błasiak, J. (2026) https://BioRender.com/ot83ppz (accessed 14 March 2026).

Remodeling of myocardial glucose metabolism in heart failure (HF) is associated with coordinated alterations in substrate utilization, mitochondrial functions, and redox homeostasis (Figure 1) [42]. Experimental and clinical studies show that although glycolytic flux is often increased in the failing myocardium, mitochondrial oxidation of pyruvate is reduced, leading to uncoupling of glycolysis from glucose oxidation and thereby decreasing cardiac efficiency [43]. This mismatch results in greater reliance on less efficient ATP-producing pathways and contributes to the energetic deficit observed in HF, despite partial compensation at the level of cellular ATP generation [44]. Changes in substrate flux through glycolysis, TCA, and OXPHOS can modulate the NAD^+^/NADH ratio. This ratio is dynamically buffered by enzymatic systems such as lactate dehydrogenase, which interconverts pyruvate and lactate while regenerating NAD^+^, as well as by intracellular redox shuttles that equilibrate cytosolic and mitochondrial reducing equivalents [44,45,46]. Fluctuations in NAD^+^ availability directly affect sirtuins, thereby linking metabolic state to post-translational protein modification [45]. Reduced NAD^+^ levels and altered NAD^+^/NADH ratios observed in experimental models of cardiometabolic disease have been associated with impaired mitochondrial function, increased protein acylation, and diminished antioxidant capacity [47]. Restoration of NAD^+^ pools in preclinical HF models improves mitochondrial function and enhances sirtuin-dependent deacetylation pathways, supporting metabolic remodeling and stress resistance [48]. Altogether, these observations support a model in which myocardial substrate metabolism, redox state, and protein acylation create a dynamic network rather than a simple cascade. Disruption of this network contributes to the progression of cardiac remodeling and functional decline in HF.

### 2.3. Differences Across Heart Failure Phenotypes

In heart failure with reduced ejection fraction (HFrEF), advanced mitochondrial dysfunction and impaired oxidative metabolism predominate, often accompanied by marked uncoupling of glycolysis from glucose oxidation and reduced activity of mitochondrial sirtuins [49,50]. In contrast, heart failure with preserved ejection fraction (HFpEF) is characterized by metabolic inflexibility, reduced energetic reserve, and chronic low-grade inflammation, frequently occurring in the absence of overt ischemia [24]. HFpEF is strongly associated with systemic metabolic disorders, including obesity and insulin resistance, which are linked to disturbances in NAD^+^ metabolism and may disproportionately impact sirtuin-dependent signaling pathways. Diabetic HF represents a composite phenotype in which disturbances in glucose handling, FA overload, and redox imbalance converge, creating a metabolic environment particularly hostile to normal sirtuin function [42,50]. Diabetes and insulin resistance are dominant modifiers of myocardial glucose metabolism and critically shape metabolic remodeling in HF [51]. Insulin resistance limits insulin-dependent glucose uptake despite elevated circulating glucose, while basal glycolysis may remain inappropriately elevated via insulin-independent mechanisms. Concurrent suppression of glucose oxidation, driven by PDH inhibition and competition from FA oxidation, exacerbates glycolysis–oxidation uncoupling [52,53]. Chronic hyperglycemia promotes oxidative stress and DNA damage, leading to activation of NAD^+^-consuming pathways such as poly(ADP-ribose) polymerases (PARPs) and cluster of differentiation 38 (CD38), which in turn deplete intracellular NAD^+^ pools and cause sustained functional impairment of NAD^+^-dependent sirtuin activity [12,54]. In this context, sirtuins function as compromised metabolic sensors that fail to adequately coordinate glucose utilization, mitochondrial function, and stress responses, thereby contributing to the progression of diabetic cardiomyopathy and HF. However, if the protective role of certain sirtuins against HF has been reported in several studies, how can they also be described as “compromised metabolic sensors” that contribute to diabetic cardiomyopathy and HF progression? The answer is that these two views are not contradictory; they describe different functional states of the same system and will be developed in the next sections.

In summary, glucose metabolic remodeling in HF arises from a multifactorial interplay of hemodynamic stress, mitochondrial dysfunction, neurohumoral activation, and systemic metabolic disturbances. Chronic pressure or volume overload and ischemia promote a shift in substrate utilization toward increased glucose uptake and glycolysis, reflecting an adaptive response to reduced oxygen availability and impaired mitochondrial oxidative capacity. At the same time, mitochondrial dysfunction and oxidative stress suppress glucose oxidation, in part through altered regulation of the PDH complex, including increased inhibitory phosphorylation mediated by PDH kinases. This uncouples glycolysis from glucose oxidation, a hallmark of the failing myocardium. In addition, systemic factors such as insulin resistance and elevated circulating fatty acids further impair metabolic flexibility by inhibiting PDH and promoting preferential FA utilization, thereby restricting efficient glucose oxidation. Inflammation, neurohumoral activation, and altered intracellular signaling pathways further contribute to metabolic reprogramming by modifying gene expression, enzyme activity, and redox balance. Collectively, these processes drive a shift from tightly coupled oxidative metabolism to a less efficient, more heterogeneous metabolic state, which contributes to contractile dysfunction and adverse cardiac remodeling.

## 3. Diabetes and Insulin Resistance as Determinants of Cardiac Glucose Metabolism: The Role of Sirtuins

Diabetes mellitus and insulin resistance are potent determinants of myocardial energy metabolism and profoundly reshape cardiac glucose utilization across various heart diseases [55]. In the healthy heart, insulin signaling promotes glucose uptake and oxidation, enabling metabolic flexibility in response to feeding, workload, and substrate availability [56]. In contrast, insulin resistance disrupts this tightly regulated balance, leading to maladaptive changes in glucose handling that directly contribute to energetic inefficiency and cardiac dysfunction [57].

A hallmark of the diabetic heart is impaired insulin-dependent glucose uptake, driven by reduced translocation and activity of the glucose transporter 4 (GLUT4) [58]. Despite elevated circulating glucose levels, cardiomyocytes become functionally glucose-restricted, limiting the contribution of glucose oxidation to mitochondrial ATP production [59]. Paradoxically, basal or stress-induced glycolytic flux may be preserved or even increased through insulin-independent mechanisms, including activation of hypoxia-responsive and inflammatory pathways [60]. This mismatch among glucose uptake, glycolysis, and downstream mitochondrial oxidation represents a central metabolic defect in diabetes-associated HF [61].

Activation of GLUT1 (SLC2A1) in the heart is crucial for metabolic reprogramming, particularly under stress conditions such as ischemia, hypertrophy, or regeneration [62]. In these contexts, increased GLUT1-driven glucose uptake supports ATP production and helps maintain mitochondrial function, contributing to cardioprotection. Within the sirtuin–GLUT axis, particularly involving SIRT1, GLUT1 acts as a downstream effector of energy-sensing pathways [63]. SIRT1, an NAD^+^-dependent deacetylase, influences cardiac metabolism by regulating transcriptional coactivators such as PGC-1α and by interacting with AMPK and FOXO signaling pathways, thereby enhancing metabolic flexibility and adaptation to energetic stress [64]. Functionally, activating SIRT1 promotes a shift toward efficient energy use and stress resistance, which may include increased glucose uptake (indirectly via GLUT1) during periods of heightened energetic demand. Conversely, higher GLUT1 activity increases intracellular glucose flux, feeding into metabolic and redox pathways that intersect with processes controlled by SIRT1, such as mitochondrial function, oxidative stress management, and autophagy [65].

**Figure 2:**
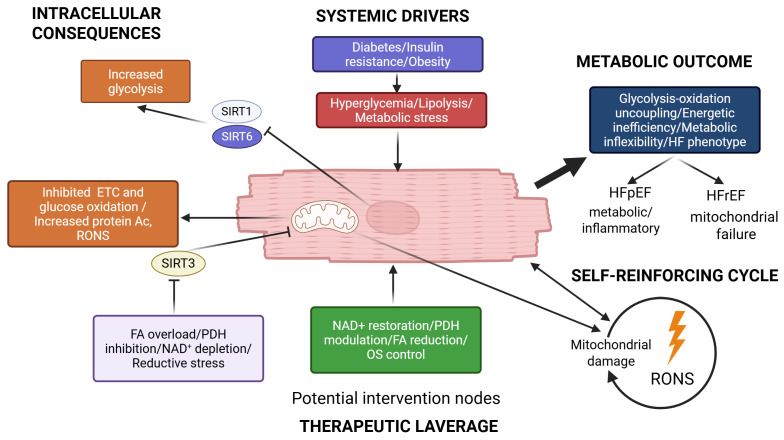
Interplay between glucose metabolism, diabetes, sirtuins, and heart failure. Integrated mechanisms linking diabetes-associated metabolic disturbances to cardiac dysfunction through sirtuin-mediated regulation. Systemic drivers such as insulin resistance, hyperglycemia, and obesity increase fatty acid (FA) delivery, promote oxidative stress (OS), and activate oxidized nicotinamide dinucleotide (NAD^+^)-consuming pathways, resulting in reduced intracellular NAD^+^ availability, suppressing NAD^+^-dependent sirtuins. Mitochondrial SIRT3 regulates oxidative metabolism and antioxidant defenses within the mitochondrial matrix; its impairment leads to mitochondrial protein hyperacetylation, reduced electron transport chain (ETC) efficiency, increased reactive oxygen and nitrogen species (RONS), and decreased glucose oxidation. Nuclear sirtuins (SIRT1 and SIRT6) modulate transcriptional programs controlling glycolysis, mitochondrial biogenesis, and stress responses, and their dysfunction contributes to increased glycolytic flux and metabolic imbalance. Concurrent inhibition of pyruvate dehydrogenase (PDH) further suppresses glucose oxidation. These processes converge to produce uncoupling of glycolysis from mitochondrial glucose oxidation, energetic inefficiency, and metabolic inflexibility, which contribute to the development of HF phenotypes: HF with preserved ejection fraction (HFpEF) and HF with reduced ejection fraction (HFrEF). A self-reinforcing cycle emerges in which mitochondrial dysfunction and RONS generation amplify redox imbalance and further impair sirtuin signaling. Potential therapeutic strategies target multiple nodes within this network, including NAD^+^ restoration, modulation of PDH activity, reduction in FA overload, and improvement of mitochondrial function, emphasizing restoration of metabolic coordination rather than single-pathway intervention. Created in BioRender. Błasiak, J. (2026) https://BioRender.com/yvp0hmx (accessed 14 March 2026).

At the mitochondrial level, suppression of glucose oxidation is largely driven by inhibition of pyruvate dehydrogenase (PDH), the rate-limiting enzyme linking glycolysis to the tricarboxylic acid cycle [66] (Figure 2). In insulin-resistant states, PDH activity is reduced by increased expression and activity of pyruvate dehydrogenase kinases, as well as by substrate competition from elevated fatty acid oxidation. Increased delivery and oxidation of fatty acids raise mitochondrial acetyl-CoA and NADH levels, which feed back to inhibit PDH according to the principles of the glucose–fatty acid (Randle) cycle [67]. As a result, glycolytically derived pyruvate is diverted toward lactate production rather than complete oxidation, thereby exacerbating the uncoupling of glycolysis from glucose oxidation.

These alterations in glucose metabolism have significant consequences for myocardial energetics and redox balance. Glycolysis alone provides insufficient ATP to meet the heart’s continuous energy demands, while excessive reliance on fatty acid oxidation increases oxygen consumption and production of reactive oxygen and nitrogen species (RONS) [68]. The diabetic myocardium, therefore, operates in a state of relative energetic compromise, marked by reduced ATP efficiency, oxidative stress, and impaired metabolic flexibility. Over time, this metabolic milieu contributes to mitochondrial dysfunction, contractile impairment, and adverse remodeling.

Importantly, the impact of diabetes on cardiac glucose metabolism varies across HF phenotypes [69]. In diabetic cardiomyopathy and HFpEF, suppression of glucose oxidation and fatty acid overload coexist with relatively preserved systolic function but marked energetic and diastolic impairment. In HFrEF, insulin resistance and hyperglycemia further exacerbate an already compromised oxidative capacity, reinforcing metabolic inflexibility and accelerating disease progression. In both settings, diabetes acts as a powerful modifier of myocardial metabolism rather than a simple comorbidity. Collectively, diabetes and insulin resistance reshape cardiac glucose metabolism by limiting insulin-mediated glucose utilization, promoting uncoupling of glycolysis and OXPHOS, and inducing a redox environment unfavorable to adaptive metabolic signaling. These changes position glucose metabolism as a central mechanistic link between systemic metabolic disease and HF, highlighting the importance of targeting metabolic pathways to improve outcomes in diabetic heart disease.

In the diabetic and insulin-resistant heart, dysregulated glucose metabolism can be viewed as a failure of the NAD^+^–sirtuin–PDH regulatory axis, which normally coordinates glycolytic flux with mitochondrial glucose oxidation and stress adaptation [70]. As mentioned, chronic hyperglycemia and fatty acid overload increase mitochondrial reducing pressure and oxidative stress, activating NAD^+^-consuming pathways that deplete intracellular NAD^+^ pools. Reduced NAD^+^ availability suppresses sirtuin activity, particularly mitochondrial SIRT3 and, indirectly, nuclear SIRT1 and SIRT6, leading to hyperacetylation of key metabolic enzymes and transcriptional regulators. Within mitochondria, diminished SIRT3 activity impairs deacetylation and the optimal function of enzymes involved in the TCA cycle, electron transport, and antioxidant defense, thereby reducing oxidative capacity and amplifying redox imbalance [71]. Concurrently, PDH activity is suppressed by inhibitory phosphorylation by PDH kinases and by substrate-driven feedback inhibition due to elevated fatty acid oxidation. Loss of sirtuin-mediated coordination exacerbates this state by weakening transcriptional and post-translational control over PDH and related pathways, reinforcing the uncoupling of glycolysis from glucose oxidation. As a result, glycolytically derived pyruvate is preferentially converted to lactate rather than oxidized, ATP efficiency declines, and metabolic flexibility is lost. Therefore, diabetes does not merely alter substrate availability but destabilizes a core epigenetic–metabolic sensing network. NAD^+^ depletion converts normally protective sirtuin signaling into a state of functional insufficiency, thereby driving energetic failure and accelerating the progression of diabetic cardiomyopathy and HF.

The role of sirtuins in the molecular mechanisms of HF pathogenesis is summarized in Table 1.

## 4. Sirtuin-Mediated Regulation of Glucose Metabolism in Diabetic Heart Failure

Diabetic HF, also called diabetic cardiomyopathy, is a distinct cardiometabolic entity in which chronic hyperglycemia, insulin resistance, and altered substrate availability fundamentally reshape myocardial energy metabolism [72]. In this setting, sirtuins play a critical regulatory role at the interface of glucose handling, mitochondrial function, and redox homeostasis [49,73]. Although sirtuins are intrinsically cardioprotective, the diabetic metabolic environment progressively compromises their activity, contributing to the characteristic metabolic inflexibility and impaired glucose oxidation observed in diabetic HF [74].

### 4.1. Nuclear Sirtuins and Transcriptional Dysregulation of Glucose Utilization

In the diabetic heart, nuclear sirtuins such as SIRT1 and SIRT6 play key roles in regulating the transcription of genes involved in glucose metabolism, yet their function is strongly constrained by NAD^+^ depletion and oxidative stress [75]. SIRT1 normally promotes metabolic adaptability by modulating transcription factors, including peroxisome proliferator-activated receptor-γ coactivator-1α (PGC-1α), forkhead box O (FOXO) proteins, and nuclear factor kappa B (NF-κB), thereby influencing insulin signaling, mitochondrial biogenesis, and antioxidant capacity [76]. Under chronic hyperglycemia and insulin resistance, however, reduced NAD^+^ availability and persistent inflammatory signaling limit SIRT1 activity. This blunts transcriptional programs that favor efficient glucose oxidation and stress resistance, shifting the myocardium toward a less adaptable metabolic state.

SIRT6 exerts a more direct control over glycolytic flux by repressing transcription of genes encoding key glycolytic enzymes through histone deacetylation [77]. In the non-diabetic myocardium, this repression helps balance glycolysis with mitochondrial oxidation. In diabetic HF, functional suppression of SIRT6 represses glycolytic pathways, thereby increasing glycolytic flux despite impaired downstream oxidation [25]. This transcriptional imbalance contributes to excessive reliance on glycolysis in the absence of efficient glucose oxidation, reinforcing one of the central metabolic defects of the diabetic failing heart.

### 4.2. Mitochondrial Sirtuins and Impaired Glucose Oxidation in Diabetes

Mitochondrial SIRT3 is the principal sirtuin isoform regulating oxidative metabolism in the heart and is particularly relevant in diabetic HF [78]. SIRT3 deacetylates and activates enzymes involved in TCA, ETC, and RONS detoxification, thereby supporting efficient mitochondrial utilization of glucose-derived substrates [79]. In diabetes, however, chronic nutrient excess, fatty acid overload, and oxidative stress deplete mitochondrial NAD^+^ pools, thereby functionally suppressing SIRT3 activity.

Loss of SIRT3 function promotes widespread mitochondrial protein hyperacetylation, impairing OXPHOS and antioxidant defenses. Consequently, even when glycolysis supplies pyruvate, the diabetic mitochondrion has a reduced capacity to fully oxidize glucose-derived acetyl-CoA [79]. This defect is particularly detrimental in diabetic HF, where mitochondrial inefficiency and oxidative stress coexist with preserved or increased glycolytic flux, thereby worsening energetic mismatch and contractile dysfunction [54].

### 4.3. Sirtuins, Pyruvate Dehydrogenase Regulation, and Glycolysis–Oxidation Uncoupling in Diabetic Heart Failure

A central metabolic hallmark of diabetic HF is the uncoupling of glycolysis from glucose oxidation, and sirtuin signaling plays an important integrative role in this process. Pyruvate dehydrogenase, the rate-limiting enzyme linking cytosolic glycolysis to mitochondrial glucose oxidation, is strongly inhibited in diabetes due to increased activity of PDH kinases and substrate competition from elevated fatty acid oxidation [80]. Sirtuin dysfunction exacerbates this inhibition by destabilizing the mitochondrial redox and acetylation environment that normally supports PDH activity [81,82].

Under conditions of reduced NAD^+^ and diminished SIRT3 function, mitochondrial hyperacetylation and oxidative stress further suppress PDH-dependent glucose oxidation [83]. Concurrent nuclear sirtuin impairment weakens transcriptional control of metabolic flexibility, limiting compensatory responses. The combined effect is a persistent state in which glycolysis proceeds independently of mitochondrial oxidation, leading to lactate accumulation, reduced ATP efficiency, and increased metabolic stress. This glycolysis–oxidation uncoupling is especially pronounced in diabetic HF, where hyperglycemia and insulin resistance amplify each component of the dysregulated network [84].

### 4.4. Implications for Diabetic Heart Failure Phenotypes and Therapy

The impact of sirtuin dysregulation on glucose metabolism is particularly relevant to diabetic cardiomyopathy and HFpEF, in which metabolic and energetic impairment often precedes overt systolic dysfunction [85]. In HFrEF, diabetes further exacerbates mitochondrial failure and limits residual adaptive capacity [52]. Across these phenotypes, impaired sirtuin signaling emerges not as a primary genetic defect but as a secondary consequence of metabolic overload and redox imbalance. Therefore, these observations identify the NAD^+^–sirtuin axis as a central mechanistic link among diabetes, impaired glucose metabolism, and HF. Restoring NAD^+^ availability or selectively enhancing sirtuin activity has therefore attracted interest as a therapeutic strategy to recouple glycolysis and glucose oxidation, improve mitochondrial efficiency, and mitigate energetic failure in diabetic HF, although effective clinical translation remains a challenge.

In non-diabetic HF, alterations in glucose metabolism largely reflect secondary adaptations to various stresses, with increased reliance on glycolysis often compensating for impaired oxidative capacity [86]. In contrast, diabetic HF is characterized by a primary metabolic derangement driven by insulin resistance, chronic hyperglycemia, and FA overload, which suppress glucose oxidation even when glycolytic flux is preserved or elevated [15]. This diabetic metabolic milieu is marked by a persistent redox imbalance and NAD^+^ depletion, impairing sirtuin-mediated metabolic sensing and coordination. As a result, while sirtuin signaling may remain partially adaptive in non-diabetic HF, diabetic HF exhibits a more profound and sustained failure of the NAD^+^–sirtuin axis, exacerbating glycolysis–oxidation uncoupling, energetic inefficiency, and disease progression.

## 5. Experimental and Clinical Evidence of the Sirtuin–Diabetes–Glucose Metabolism Interplay in Heart Failure

Evidence linking sirtuin signaling to cardiac glucose metabolism in the context of HF and diabetes has emerged across multiple experimental levels, including genetically modified animal models, cellular studies, and human clinical observations. These data support a mechanistically coherent yet context-dependent role for sirtuins as metabolic regulators, with their protective capacity progressively constrained by diabetic metabolic stress.

### 5.1. Evidence from Genetically Modified Mouse Models

Mouse models in which sirtuin-encoding genes are manipulated have provided mechanistic insights into the role of sirtuins in cardiac metabolism and HF progression [87]. Cardiac-specific deletion of the *SIRT3* gene results in widespread hyperacetylation of mitochondrial proteins, impaired OXPHOS, increased RONS production, and accelerated development of cardiac hypertrophy and dysfunction, particularly under metabolic stress [88]. In models of obesity or diabetes, *SIRT3* deficiency exacerbates suppression of glucose oxidation and mitochondrial inefficiency, supporting a causal role for SIRT3 in maintaining oxidative glucose metabolism and redox balance [89].

Conversely, gain-of-function models and pharmacological enhancement of the NAD^+^–SIRT3 axis improve mitochondrial function, restore metabolic flexibility, and attenuate cardiac dysfunction in diabetic and pressure-overload heart-failure models [90,91]. Similar trends have been reported for SIRT1, where moderate cardiac overexpression confers protection against oxidative stress and metabolic dysfunction, whereas excessive or prolonged activation can be maladaptive, highlighting dose- and context-dependent effects [92].

Importantly, mouse models of cardiometabolic HF have shown that NAD^+^ depletion precedes overt systolic impairment and that restoring NAD^+^ availability improves cardiac metabolic efficiency in a sirtuin-dependent manner [93,94]. These findings provide experimental support for the concept that diabetes-associated HF reflects, at least in part, a state of functional sirtuin insufficiency driven by disrupted NAD^+^ homeostasis.

### 5.2. Insights from In Vitro Studies

In vitro studies using primary cardiomyocytes and cardiac-derived cell lines have further clarified the molecular mechanisms linking sirtuins to glucose metabolism. Under high-glucose or lipid-overload conditions, cardiomyocytes show reduced NAD^+^ levels, increased mitochondrial protein acetylation, and impaired oxidative metabolism, along with suppressed SIRT1 and SIRT3 activity [95,96]. Experimental restoration of NAD^+^ or direct activation of sirtuins in these systems enhances mitochondrial respiration, reduces oxidative stress, and promotes the oxidative utilization of glucose-derived substrates.

Cellular studies have also shown that sirtuins indirectly influence PDH function by modulating mitochondrial redox and acetylation states [71]. Loss of SIRT3 activity favors PDH inhibition and reinforces the uncoupling of glycolysis and oxidation, whereas restoration of sirtuin signaling partially rescues mitochondrial glucose oxidation [10]. Nuclear sirtuins, particularly SIRT6, restrain excessive glycolysis by repressing the transcription of glycolytic genes, suggesting that sirtuin dysfunction contributes to both transcriptional and post-translational dysregulation of glucose metabolism in diabetes [10].

While these cellular findings offer strong mechanistic support, in vitro systems capture only acute or simplified aspects of metabolic stress and cannot fully reproduce the chronic, multisystem nature of diabetic HF.

### 5.3. Human Myocardial and Circulating Biomarker Evidence

Human data, though limited, provide translational relevance to experimental findings. Analyses of myocardial tissue from patients with HF, especially those with metabolic comorbidities, have identified reduced myocardial NAD^+^ levels, increased mitochondrial protein acetylation, and altered expression or activity of key sirtuin isoforms [97,98,99]. Although direct measurements of sirtuin enzymatic activity in human hearts are scarce, patterns of protein acetylation and redox imbalance are consistent with impaired sirtuin function.

Circulating biomarkers further support this link. Reduced systemic NAD^+^ metabolites, altered nicotinamide metabolism, and increased markers of oxidative stress have been reported in patients with diabetes and HF [100]. These biomarker associations with adverse cardiac outcomes suggest a clinically relevant disruption of the NAD^+^–sirtuin axis. Moreover, early-phase clinical studies of NAD^+^ precursors have shown improvements in systemic metabolic parameters and mitochondrial function, although definitive cardiac endpoints remain under study [101]. Notably, human studies also show substantial heterogeneity. Differences in heart-failure phenotype, diabetes duration, glycemic control, age, and comorbidities complicate interpretation and underscore the multifactorial nature of sirtuin dysregulation in human disease.

### 5.4. Limitations

Despite strong experimental support, several contradictions and limitations warrant critical appraisal. First, as mentioned above, sirtuin expression and function do not always correlate: in both animal and human studies, sirtuin protein levels may be preserved or even increased, whereas enzymatic activity is reduced due to NAD^+^ depletion. This distinction complicates the interpretation of expression-based analyses and may contribute to apparently conflicting results. Second, sirtuin effects are highly context-dependent. While sirtuin activation is protective during early or moderate metabolic stress, excessive activation or late-stage intervention may be ineffective or even detrimental, reflecting the narrow adaptive window of these pathways [102]. Third, extrapolation from animal models to humans remains imperfect because rodent metabolism, disease course, and response to intervention differ substantially from those in patients with chronic diabetes and HF [103]. Finally, human clinical evidence remains mostly associative. Direct evidence that restoring sirtuin activity improves cardiac glucose metabolism and outcomes in diabetic heart failure is still lacking, underscoring the need for longitudinal, tissue-specific, and mechanism-focused clinical studies.

A comparison of experimental and clinical evidence from studies on the interplay among sirtuins, diabetes, and cardiac glucose metabolism in HF is presented in Table 2. Taken together, experimental and clinical evidence supports a model in which sirtuins serve as central regulators of cardiac glucose metabolism, with their activity progressively constrained by diabetes-induced NAD^+^ depletion and redox stress. While animal and cellular studies provide compelling mechanistic proof of principle, human data highlight complexity, heterogeneity, and important translational gaps. This body of evidence suggests that sirtuin dysfunction is not merely an epiphenomenon but a mechanistically relevant contributor to metabolic inflexibility in diabetic HF, albeit one whose therapeutic exploitation requires consideration of timing, phenotype, and metabolic context.

## 6. Therapeutic Implications

The converging experimental and clinical evidence linking sirtuin signaling with altered glucose metabolism in diabetic heart failure has important therapeutic implications. Rather than identifying a single actionable target, this body of work reframes diabetic heart failure as a disorder of metabolic sensing and coordination, in which disruption of the NAD^+^–sirtuin axis amplifies glycolysis–oxidation uncoupling, mitochondrial dysfunction, and energetic inefficiency. Therapeutic strategies emerging from this framework, therefore, aim not merely to modify substrate availability but to restore metabolic flexibility and redox-sensitive regulation.

### 6.1. Targeting NAD^+^ Homeostasis and Sirtuin Function

Restoring NAD^+^ availability is a central therapeutic strategy for targeting sirtuins in diabetic HF. Preclinical studies show that NAD^+^ repletion can improve mitochondrial efficiency, reduce protein hyperacetylation, and partially uncouple glucose oxidation from glycolysis in cardiometabolic HF models [100]. This suggests that NAD^+^-boosting strategies may enhance residual sirtuin activity and restore adaptive metabolic signaling. However, current human data remain limited, and whether such approaches can improve cardiac outcomes, particularly in advanced disease, remains to be established.

These and other findings underscore that direct sirtuin activation may be context-dependent [104]. While sirtuin activation is protective during early or moderate metabolic stress, advanced diabetic HF is marked by a profound redox imbalance and mitochondrial dysfunction, conditions in which sirtuin pathways may be less responsive [105]. Therapeutic efforts may therefore need to focus on upstream metabolic correction, such as reducing oxidative stress and restoring NAD^+^ balance, rather than solely on activating sirtuins.

### 6.2. Modulating Glucose Oxidation and Metabolic Coupling

The connection among sirtuin dysfunction, PDH inhibition, and the uncoupling of glycolysis from oxidation underscores glucose oxidation as a potential therapeutic target. In diabetic HF, directly increasing glucose supply or uptake would probably not improve energy efficiency if mitochondrial oxidation remains suppressed [106]. Instead, therapies that reduce PDH inhibition or enhance mitochondrial oxidative capacity may be necessary for effective metabolic recovery [107]. From this viewpoint, sirtuin signaling acts as a key regulator of treatment success: without an adequate NAD^+^–sirtuin environment, interventions that modify substrate flux may have limited impact.

This framework clarifies why traditional metabolic interventions often show inconsistent results in diabetic HF. Treatments that improve overall blood sugar management or reduce fatty acid overload can indirectly support cardiac metabolism by reducing redox stress and NAD^+^ depletion. This helps restore the heart’s natural ability to adapt its metabolism, rather than forcing a particular substrate choice.

### 6.3. Phenotype Specificity and Time Dependence

A critical implication of the sirtuin–metabolism interplay is that therapeutic efficacy likely depends on the HF phenotype, disease stage, and metabolic context. In diabetic cardiomyopathy and HFpEF, conditions marked by early energetic impairment and metabolic inflexibility, restoring sirtuin-mediated coordination may be particularly relevant. In contrast, in advanced HFrEF, where mitochondrial oxidative capacity is severely compromised, sirtuin-targeted strategies alone may be insufficient without concurrent improvement in mitochondrial structure and function.

Timing also appears critical. Experimental data suggest a narrow window during which modulation of sirtuin signaling is beneficial, whereas late intervention may be ineffective or even maladaptive [95,96,108]. This reinforces the concept that therapies emerging from this framework are best viewed as disease-modifying rather than symptomatic, with the greatest potential impact when applied before irreversible energetic failure is established.

### 6.4. Integration with Existing Therapies and Precision Medicine: Limitations

The sirtuin-centric view of diabetic HF does not replace established hemodynamic or neurohormonal therapies but complements them by offering a metabolic rationale for the heterogeneity in treatment response. Variability in redox state, NAD^+^ metabolism, and sirtuin function may contribute to differential outcomes among patients with otherwise similar clinical profiles [109]. In this sense, once validated, sirtuin-related biomarkers could inform metabolic phenotyping and precision-based therapeutic strategies, guiding the selection and timing of interventions that target metabolic inefficiency.

Despite a compelling mechanistic rationale, the translation of sirtuin-based strategies into clinical practice remains limited by incomplete human evidence. Direct assessment of myocardial sirtuin activity, NAD^+^ compartmentalization, and glucose metabolic fluxes in patients is challenging, and current clinical trials have primarily focused on systemic metabolic endpoints rather than cardiac-specific outcomes [110]. Future therapeutic development will require integrative approaches that combine metabolic imaging, biomarker profiling, and longitudinal assessment to determine whether restoring the NAD^+^–sirtuin axis yields durable improvements in cardiac function and prognosis in diabetic HF.

Overall, the therapeutic implications of the interplay among sirtuins, diabetes, HF, and glucose metabolism emphasize metabolic restoration rather than single-pathway correction. Table 3 presents established and conceptual therapeutic strategies applicable to diabetic HF. By situating sirtuins within a broader network of redox balance, mitochondrial function, and substrate utilization, this framework points toward combination and timing-sensitive strategies aimed at restoring the heart’s capacity for adaptive energy metabolism. Although clinical translation remains in its early stages, this perspective provides a biologically coherent foundation for future therapeutic innovation in diabetic HF.

In addition to their well-established intracellular roles, sirtuins have been detected in circulation, including in plasma, serum, and peripheral blood mononuclear cells [111]. Circulating levels of selected sirtuins, particularly SIRT1 and SIRT3, have been associated with HF, suggesting their potential utility as biomarkers for this disease [12,112]. However, their precise biological function in the extracellular milieu and their direct contribution to HF pathogenesis remain incompletely understood, and current data are largely correlative. Circulating sirtuins have recently emerged as potential noninvasive biomarkers (“liquid biopsy”) that reflect systemic metabolic and vascular stress [113]. Alterations in circulating SIRT1 and SIRT3 levels have been associated with heart failure, oxidative stress, and endothelial dysfunction. Notably, preliminary evidence suggests that circulating sirtuins may reflect differences in underlying pathophysiological mechanisms across HF phenotypes, such as HFpEF, predominantly characterized by metabolic and microvascular dysfunction, and HFrEF, more closely linked to cardiomyocyte loss and contractile impairment [114]. Moreover, circulating sirtuins may serve as early indicators of major adverse cardiac events (MACEs), remodeling, and increased cardiovascular risk [115]. However, these observations remain largely correlative and require further validation before clinical implementation.

## 7. Conclusions, Perspectives, and Outstanding Questions

Accumulating experimental and clinical evidence supports a central role for sirtuins as metabolic and redox-sensitive regulators that integrate glucose metabolism, mitochondrial function, and stress responses in the failing heart. In diabetes, chronic hyperglycemia and insulin resistance impose a sustained metabolic burden that disrupts NAD^+^ homeostasis, suppressing sirtuin activity and impairing adaptive control of glucose utilization. This disruption contributes to hallmark features of diabetic HF, including uncoupling of glycolysis and oxidation, mitochondrial inefficiency, oxidative stress, and energetic compromise.

Sirtuin signaling in the heart is generally not considered a primary genetic defect but rather a dynamic, stress-responsive system that reflects the cell’s metabolic and redox state. As NAD^+^-dependent deacetylases, sirtuins serve as central metabolic sensors that link energy availability to mitochondrial function, oxidative stress responses, and cellular survival pathways. Under pathological conditions such as HF, diabetes, or ischemia, alterations in NAD^+^ metabolism, mitochondrial dysfunction, and chronic oxidative stress lead to secondary changes in sirtuin activity. In this context, reduced SIRT1 and SIRT3 signaling has consistently been associated with impaired mitochondrial function, increased RONS production, and adverse cardiac remodeling. Importantly, restoring sirtuin activity through pharmacological or metabolic interventions has been shown to confer cardioprotective effects, supporting the notion that sirtuin dysregulation is primarily an adaptive, potentially maladaptive response to cellular stress rather than a direct causative genetic abnormality [116,117].

Importantly, sirtuins emerge not as isolated molecular targets but as nodes within a broader regulatory network linking substrate flux, redox balance, and epigenetic control. Their dual characterization as intrinsically cardioprotective yet functionally insufficient under diabetic metabolic stress reconciles seemingly contradictory experimental findings and underscores the importance of metabolic context, disease stage, and NAD^+^ availability. Collectively, these insights reframe diabetic HF as a disorder of impaired metabolic sensing and coordination.

Looking ahead, the sirtuin–NAD^+^ axis provides a conceptual framework for understanding heterogeneity in metabolic remodeling and treatment response across HF phenotypes. Rather than implying uniform benefit, this perspective emphasizes that interventions aimed at restoring sirtuin signaling are likely to be phenotype-specific, timing-dependent, and context-sensitive. Early diabetic cardiomyopathy and HFpEF, characterized by metabolic inflexibility and preserved contractile reserve, may represent particularly relevant windows for targeting metabolic coordination. In contrast, advanced HFrEF with extensive mitochondrial damage may require combined strategies that address structural, oxidative, and metabolic deficits.

From a translational perspective, this framework also underscores the value of metabolic and redox biomarkers that reflect NAD^+^ status, protein acetylation, or sirtuin activity, rather than relying solely on gene expression. These markers could enable metabolic phenotyping of patients, inform timing of therapy, and guide the rational integration of metabolic strategies with established hemodynamic and neurohormonal therapies. Ultimately, the sirtuin-centric view supports a shift toward precision cardiometabolic medicine, in which restoring adaptive metabolic capacity complements conventional heart-failure management.

As mentioned, circulating SIRT1 and SIRT3 levels may correlate with disease severity and prognosis in cardiometabolic disorders, including HF. This raises the possibility that circulating sirtuins could inform patient risk stratification and monitoring of disease progression. Furthermore, because sirtuins function as NAD^+^-dependent metabolic sensors, their activity may be linked to responses to NAD^+^-boosting strategies; however, this concept remains largely hypothetical and requires further clinical validation.

Several key questions remain unresolved in the interplay between sirtuins, HF, diabetes and glucose metabolism, setting priorities for future research. An important issue that should be addressed in future research is causality in humans: To what extent does reduced sirtuin activity cause, rather than just associate with, metabolic dysfunction in human diabetic HF? Can this be addressed in cardiac-specific interventional studies? As NAD+ is an essential link in this interplay, the problem of NAD+ compartmentalization should be further explored to elucidate how changes in NAD+ pools across the nuclear, cytosolic, and mitochondrial compartments differentially influence sirtuin function and glucose metabolism in the diabetic heart. The pronounced functional differences between HF phenotypes raise the question of whether HFrEF and HFpEF exhibit distinct patterns of sirtuin dysregulation and metabolic vulnerability in the presence of diabetes. Temporal dynamics matter for therapy. Therefore, it is important to determine at what stage of diabetic HF restoring sirtuin signaling retains therapeutic potential, and when irreversible mitochondrial damage renders it ineffective. Also, the problem of unwanted side effects should be addressed alongside its efficacy across all kinds of therapies; therefore, the outstanding question is how sirtuin-oriented metabolic strategies can be combined with existing therapies to maximize benefit without causing unintended metabolic or redox imbalances. Finally, is it possible to develop reliable, clinically accessible biomarkers of myocardial sirtuin activity or NAD^+^ levels to aid patient stratification and treatment monitoring?

In summary, the interplay among sirtuins, diabetes, HF, and glucose metabolism reveals a unifying mechanistic narrative in which failure of NAD^+^-dependent metabolic sensing links systemic metabolic disease to cardiac energetic dysfunction. Although experimental data provide compelling mechanistic support, translation to clinical practice will require careful consideration of context, timing, and phenotype-specific effects. Addressing these challenges holds promise not only for improving outcomes in diabetic HF but also for advancing a broader paradigm of metabolism-informed cardiovascular therapy.

## Figures and Tables

**Table 1:** Role of sirtuins in metabolic/molecular pathways in heart failure ([30,55,56,57]).

Sirtuin	Localization	Major Metabolic and Molecular Roles	Role in Heart Failure
SIRT1	Nucleus/cytoplasm	Energy sensing; regulates glucose and lipid metabolism; controls oxidative stress, inflammation, autophagy (PGC-1α ^1^, FOXO, NF-κB)	Protective context-dependent: ↓ oxidative stress, ↓ apoptosis, ↓ inflammation; excess → hypertrophy/remodeling risk
SIRT2	Cytoplasm	Cytoskeleton regulation, glycolysis, cell cycle	Unclear: limited and inconsistent evidence; no well-defined HF phenotype
SIRT3	Mitochondria	Mitochondrial metabolism: FA oxidation, TCA cycle, RONS neutralization	Protective: ↓ mitochondrial dysfunction, ↓ RONS, ↓ hypertrophy and fibrosis; prevents HF progression
SIRT4	Mitochondria	Inhibits FA oxidation; regulates mitochondrial metabolism	Likely detrimental: ↑ metabolic inflexibility, ↑ hypertrophic signaling; may worsen HF
SIRT5	Mitochondria	Controls metabolic flux via desuccinylation; ammonia and redox metabolism	Protective (emerging): supports metabolic adaptation; may attenuate HF under stress
SIRT6	Nucleus	Suppresses glycolysis; regulates inflammation and DNA repair	Protective: ↓ hypertrophy, ↓ fibrosis, ↓ inflammation; preserves cardiac function
SIRT7	Nucleus (nucleolus)	Ribosome biogenesis, stress resistance, mtQC	Protective: prevents cardiomyopathy, apoptosis, and inflammatory remodeling

^1^ Acronyms: PGC-1α, peroxisome proliferator-activated receptor-γ coactivator-1α; FOXO, forkhead box O; NF-κB, nuclear factor kappa B; HF, heart failure; FA, fatty acid; TCA, tricarboxylic acid; RONS, reactive oxygen and nitrogen species; mtQC, mitochondrial quality control.

**Table 2:** Experimental and clinical evidence linking sirtuins, diabetes, and cardiac glucose metabolism in heart failure.

Aspect	Experimental Evidence	Clinical Evidence
Primary models	Genetically modified mice (*SIRT ^a^* KO), diet-induced obesity/diabetes models, pressure-overload HF models; isolated cardiomyocytes under high glucose or lipid conditions	Observational cohorts of diabetic and non-diabetic HF patients; myocardial biopsies or explanted hearts; circulating biomarkers
Control of variables	High level of experimental control over genetics, diet, timing, and metabolic stress	Limited control; high heterogeneity in comorbidities, medications, and disease duration
Sirtuin manipulation	Direct genetic or pharmacological manipulation; NAD+ precursor supplementation	Mostly indirect assessment of expression or acetylation; limited interventional studies
NAD+ biology	Causal link between NAD+ depletion and dysfunction; rescue by NAD+ repletion	Reduced NAD+ metabolites reported; largely associative data
Glucose metabolism	Direct measurement of glycolysis, glucose oxidation, PDH activity, metabolic flux	Inferred from metabolomics or imaging; direct flux measurements are rare
Mitochondrial function	Direct assessment of respiration, RONS, enzyme acetylation, and activity	Evidence of dysfunction and hyperacetylation; limited mechanistic resolution
Phenotype specificity	Clear stratification (diabetic vs. non-diabetic, early vs. late disease)	HF phenotypes are often mixed; diabetes severity varies
Temporal resolution	Longitudinal designs capturing disease progression	Primarily cross-sectional
Causal inference	Strong cause–effect relationships established	Predominantly correlative
Key limitations	Species differences; simplified models; supraphysiological manipulations	Limited tissue access; confounding variables; ethical constraints
Overall contribution	Defines mechanistic frameworks and biological plausibility	Provides translational relevance but incomplete validation

*^a^* Acronyms: SIRT, sirtuin; KO, knock-out; HF, heart failure; NAD^+^, nicotinamide adenine dinucleotide; PDH, pyruvate dehydrogenase; RONS, reactive oxygen and nitrogen species.

**Table 3:** Metabolic drivers of diabetic heart failure and corresponding therapeutic strategies.

Pathogenic Driver	Key Features in Diabetic HF	Pathophysiological Consequences	Established/Conceptual Interventions	Notes and Limitations
NAD^+^ depletion	Reduced intracellular NAD^+^ availability due to increased consumption (PARPs, CD38) and impaired salvage pathways	Functional suppression of NAD^+^-dependent enzymes (sirtuins), impaired redox balance, mitochondrial dysfunction	*Established*: Glycemic control, reduction of oxidative stress; Emerging: NAD^+^ precursors (e.g., nicotinamide riboside, nicotinamide mononucleotide), CD38 inhibition (experimental)	NAD^+^ restoration appears most effective in early cardiometabolic disease; limited efficacy in advanced HFrEF with irreversible mitochondrial damage
Sirtuin dysfunction (SIRT1, SIRT3, SIRT6)	Reduced activity despite preserved expression; impaired nuclear–mitochondrial metabolic coordination	Mitochondrial protein hyperacetylation, reduced glucose oxidation, impaired antioxidant defense, and transcriptional dysregulation	*Conceptual/Experimental*: NAD^+^ restoration to reenable endogenous sirtuin activity; targeted sirtuin activation (experimental)	Direct sirtuin activation is highly context- and dose-dependent; effectiveness constrained by NAD^+^ availability and mitochondrial integrity
PDH inhibition/PDK upregulation	Increased PDK expression and activity; inhibited PDH flux	Suppressed glucose oxidation, glycolysis–oxidation uncoupling, lactate accumulation	*Conceptual/Experimental*: PDK inhibition, PDH activation strategies	PDH modulation may restore glucose oxidation even in the presence of FA overload; currently limited clinical translation
FA overload	Increased FA delivery from adipose tissue; excessive FA uptake relative to oxidation	Substrate competition (Randle cycle), PDH inhibition, redox stress, lipotoxicity	Established: Weight reduction, insulin sensitization; Conceptual: Modulation of FA uptake or oxidation efficiency	Indiscriminate FA oxidation inhibition risks energetic compromise, especially in HFrEF; goal is rebalancing, not elimination
Incomplete FA oxidation	Reduced FA oxidation capacity with persistent FA influx	Accumulation of acyl-carnitines, excess NADH, mitochondrial ROS	Conceptual: Improve mitochondrial efficiency and coupling rather than suppress FAO	Particularly relevant in advanced HFrEF
Redox imbalance/oxidative stress	Elevated mitochondrial ROS, increased NADH/NAD^+^ ratio	Damage to mitochondrial enzymes, further NAD^+^ depletion, energetic inefficiency	*Established*: Indirect reduction via metabolic control; *Conceptual*: Target upstream sources of redox stress	Antioxidant monotherapy has shown limited benefit; redox imbalance reflects system-level dysfunction
Mitochondrial dysfunction	Impaired ETC flux, reduced ATP production, altered dynamics	Energetic failure, reduced contractile reserve	*Indirect*: Restore metabolic coordination (NAD^+^, PDH, FA balance); Experimental: Mitochondrial quality-control modulation	Mitochondrial damage limits reversibility in late-stage disease
Systemic insulin resistance	Impaired glucose uptake; increased adipose lipolysis	Glucose restriction, FA overload, metabolic inflexibility	*Established*: Lifestyle interventions, insulin sensitization	Most significant in HFpEF
Low-grade inflammation (especially HFpEF)	Adipokine and cytokine signaling	Impaired insulin and metabolic signaling	*Indirect*: Weight loss, metabolic normalization	Acts as an amplifier rather than a primary target

Acronyms: HF, heart failure; NAD^+^, nicotinamide adenine dinucleotide; NADH, nicotinamide adenine dinucleotide, reduced; PARP, poly(ADP-ribose) polymerase; SIRT, sirtuin; CD38, cluster differentiation 38; FA, fatty acid; PDH, pyruvate dehydrogenase; PDK, pyruvate dehydrogenase kinase; ETC, electron transport chain; HFpEF, heart failure with preserved ejection fraction; HFrEF, heart failure with reduced ejection fraction.

## Data Availability

No data were created in this work.
